# Probing ultrafast foam homogenization with grating-based X-ray dark-field imaging

**DOI:** 10.1038/s41598-025-30010-8

**Published:** 2025-11-26

**Authors:** Leonard Wegert, Constantin Rauch, Stephan Schreiner, Markus Schneider, Thilo Michel, Gisela Anton, Bruno Albertazzi, Michel Koenig, Pascal Meyer, Erik Fröjdh, Aldo Mozzanica, Yang Yang, Johannes Hornung, Bernhard Zielbauer, Artem S. Martynenko, Sébastien LePape, Stefan Funk, Paul Neumayer

**Affiliations:** 1 Plasma Physics Department, GSI Helmholtzzentrum für Schwerionenforschung, Planckstraße 1, 64291 Darmstadt, Germany; 2 Erlangen Centre for Astroparticle Physics (ECAP), Friedrich-Alexander-Universität Erlangen-Nürnberg, Nikolaus-Fiebiger-Straße 2, 91058 Erlangen, Germany; 3 LULI-CNRS, CEA, Sorbonne Universités, École Polytechnique, Institut Polytechnique de Paris, 91120 Palaiseau Cedex, France; 4 Institute of Microstructure Technology, Karlsruhe Institute of Technology, Hermann-von-Helmholtz-Platz 1, 76344 Eggenstein-Leopoldshafen, Germany; 5 PSI Center for Photon Science, Forschungsstrasse 111, 5232 Villigen, Switzerland

**Keywords:** Inertial confinement fusion, Foam homogenization, Hydrodynamic simulations, Lasers, X-ray dark-field imaging, Grating-based phase-contrast, Materials science, Optics and photonics, Physics

## Abstract

Microstructured foams are emerging as a promising class of targets, with applications ranging from laser-driven particle acceleration to inertial confinement fusion. To unlock their full potential, a deeper understanding of their properties, especially the changes and behavior of the microstructure under extreme conditions, is required. While recently advancing 3D printed foam targets can be observed by X-ray radiography, the microstructure in chemically produced targets is far below the spatial resolution of conventional radiography. To overcome this limitation, we apply grating-based X-ray dark-field imaging to observe structural changes in foams that are rapidly heated by laser-accelerated proton pulses. The experimental data is compared to synthetic dark-field values obtained from hydrodynamic simulations of a simplified foam model. Both experimental and simulation results demonstrate the viability of utilizing grating-based dark-field imaging for observing microstructural changes in foam targets.

## Introduction

In laser-matter interaction experiments, microstructured materials, i.e. a binary density distribution of material and voids with micrometer or sub-micrometer feature sizes, bridge the gap between targets at solid (or liquid) density and gases. Upon laser irradiation, the material rapidly transforms into an expanding plasma, rarefying and filling the voids, eventually turning the structured sample into a homogeneous plasma with uniform average density. This allows, for example, to produce near-critical density plasmas, with a strong direct laser acceleration mechanism^1^ enhancement. Tailoring of the density profile by using carbon-nanotube foams also has been demonstrated to improve laser coupling in laser-ion acceleration experiments^2^. Arrays of parallel micro- and nanowires allow the ultra-short laser pulse to propagate deep into the structure, at average densities that would be inaccessible in a homogeneous plasma, leading to ultra-high energy densities^3^ and intense X-ray emission^4^. The large surface area of such wire arrays also leads to an increased production of suprathermal electrons and improved laser-ion acceleration^5,6^. Finally, various applications of microstructured materials are extensively discussed for inertial confinement fusion (ICF) targets. This includes the application of foams as ablator material to mitigate laser imprint^7,8^ and the usage of DT-wetted foams^9^ to reduce complexity in target production and to widen the design parameter space^10,11^.

Simulating microstructured materials in laser-matter interaction is challenging, due to the wide range of spatial and temporal scales involved. Different approaches to model the behavior of nano- and microstructured material under intense laser irradiation and in hydrodynamic simulations^12,13^ are being developed, and experiments to test their predictions are devised, e.g. measuring the equation-of-states^14^ or the propagation of ionization waves^8^.

Experimentally observing the microscopic properties of microstructured targets under extreme conditions remains a challenging task. Different techniques like X-ray spectroscopy, radiography and X-ray Thomson scattering are discussed and have been employed to probe temperature and density in foams and other porous material^15,16^. However, these approaches offer limited sensitivity to another key aspect in the use of microstructured materials in laser matter experiments: the process of homogenization, i.e. the transition of the binary density distribution towards a homogeneous plasma. Dark-field imaging, an image modality of X-ray Talbot interferometry, offers the unique ability to observe the structural features below the imaging system’s resolution limits and by that is capable of visualizing the homogenization of a foam target (as proposed in our previous work^17^). Hence, dark-field imaging promises to be an important diagnostic to obtain a better understanding of microstructured targets under extreme conditions.

Here, we report on an experiment investigating homogenization of foam targets. We use intense laser-accelerated ultra-short proton pulses to rapidly heat foam samples to temperatures above 10000 K. The evolution of the foam is then imaged by a bright, short X-ray pulse, generated with a second high-intensity laser pulse. While the rather large structures of a 3D printed foam can be directly imaged by projection, for the sub-micron stochastic structure of chemically produced foams we employ the dark-field (DF) image modality of a Talbot grating interferometer to visualize the presence of microstructures. This allows us to follow the rapid homogenization of microstructured foams, and thus to test our understanding of the material at high energy density conditions and its behavior on sub-micron and picosecond scales.

## Experimental setup

**Fig. 1:**
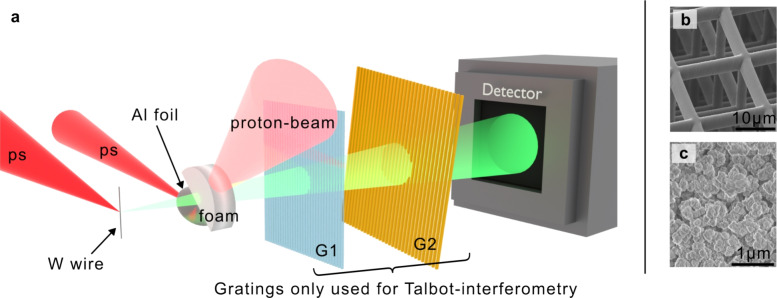
Schematic of the experimental setup (**a**). The grating setup consisting of G1 and G2 is used for Talbot-interferometry and is removable for doing standard X-ray radiography. Different types of foam targets were observed, (**b** and **c**) show scanning electron microscope pictures of their microstructure. (**b**) is a 3D printed foam with 2 μm thick rods and 20 μm spacing. (**c**) shows the much smaller structure of a chemically produced TMPTA foam. Both targets have an approximated average mass density of 250 mg/cc.

The experiment was conducted at the PHELIX facility at GSI Helmholtzzentrum für Schwerionenforschung GmbH in Germany^18^. Figure 1 shows a schematic of the experimental setup. Two high-energy laser pulses (each with an energy of approximately 30 J, at a pulse duration of 1 ps) were employed in a pump-probe configuration. The pump pulse was focused to a spot size of approximately 15 μm onto a 5 μm thin aluminum foil, producing a pulse of energetic protons via target normal sheath acceleration (TNSA)^19^. These laser-accelerated protons were used to volumetrically heat the foam samples, which were positioned 250 μm behind the foil. The probe beam was used to irradiate a thin wire target, producing a short X-ray flash for imaging. Point projection onto a 2D X-ray detector produces a magnified image of the sample, with a spatial resolution limited by the X-ray source size of approximately 5 μm. To enable dark-field imaging, a Talbot interferometer was implemented with two gratings placed between sample and X-ray detector^17,20^. By adjusting the temporal delay between the two pulses, the probe time could be varied.

Two different types of microstructured targets were used, see insets in Fig.1b and c for scanning electron microscope images. The first type of target were high-resolution 3D printed structures, produced by 2-photon-polymerization and purchased from UpNano GmbH. These targets are made out of 2 μm wide rods arranged in a cubic grid structure with a distance of 20 μm between parallel rods. By contrast, foams made of trimethylolpropane triacrylate (TMPTA) are produced by chemical methods and the targets for the experiment were acquired from Scitech Precision Ltd. These foam samples had an average density of 250 mg/cc and feature typical structure sizes well below 1 μm. Assuming typical temperatures in the order of 1 eV in the regime of warm-dense matter, the characteristic speed of expansion is given by the speed of sound ($\propto \sqrt{T}$), which is in the order of 10 km/s for eV temperatures. This yields a rough estimate of the expected homogenization times of a few hundred picoseconds for the 3D printed structures, and several tens of picoseconds for the TMPTA foams, respectively. The short duration of laser-accelerated proton pulses as well as the laser-produced X-ray source are well matched to resolve evolutions on these timescales.

**Fig. 2:**
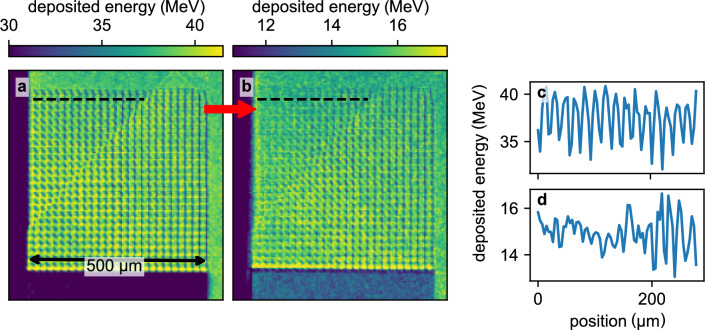
Homogenization of 3D printed foam structure after a delay of $300\,\text {ps}$. (**a**) shows the measured intensity of the cold sample as deposited X-ray energy per pixel, (**b**) shows the same sample after heating by protons. The red arrow indicates the position of the incident protons. The angled line in both images is caused by a small, non-sensitive gap in the tiled detector. The pixel values of the pixel rows indicated by the black dashed line are presented again in (**c**) and (**d**) for the cold and heated sample respectively.

## Results

### 3D-printed foam

X-ray images of the 3D printed foams are obtained directly by point projection without the use of dark-field imaging through the Talbot interferometer. Careful alignment of the samples yields a measurable attenuation by the rods parallel to the imaging axis. The rods aligned in the other directions do not stack sufficient material in imaging direction to create a visible absorption contrast. Thanks to the small X-ray source size, the periodicity of the structure is clearly resolved (Fig. 2a). When the sample is exposed to the proton pulse, the structural elements are heated and start to expand, filling the voids and thus evolving towards a homogeneous distribution. This is displayed in Fig. 2b, which shows the same sample as in **a** probed 300 ps after proton heating. Towards the top left edge, the contrast of the periodic structure is reduced, compared to the rest of the sample and the sample before heating.

The contrast and decline in contrast of the pattern can best be estimated from Fig. 2c and d, where the pixel values of single rows are plotted. In the unheated foam (Fig. 2c), the periodic structure corresponds to the 20 μm distance between parallel rods and is clearly visible over the entire pixel range. In the heated sample (Fig. 2d), the periodic structure is disturbed towards the left edge, where the heating is expected to be the strongest. The mean maximum intensity value in the unheated sample is about $39.9\,\text {MeV}$, whereas the minimum is $34.1\,\text {MeV}$, yielding a contrast of about $7.8\,\%$. The corresponding values in the left area of the heated sample are $15.3\,\text {MeV}$ and $14.4\,\textrm{MeV}$, yielding a contrast of about $3.1\,\%$. Hence, the total decrease in contrast is roughly $60\,\%$, clearly indicating a loss of structure. In the presented experiment, our limited number of 3D printed targets did not allow for a more comprehensive study including the recording of a heated foam time series. However, the results show that homogenization of larger periodic structures is observable with laser-driven X-ray backlighters, but requires care in sample alignment and magnification selection, as slight misalignments relative to the beam axis can significantly reduce contrast.

**Fig. 3:**
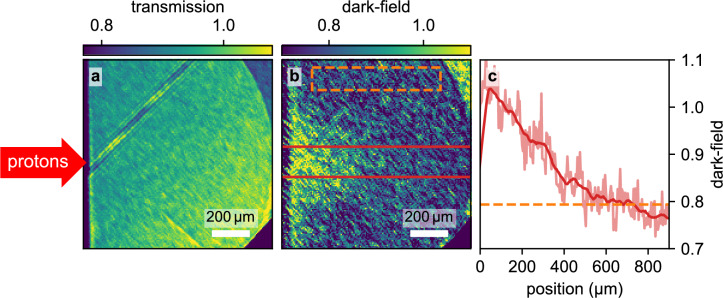
X-ray Transmission (**a**) and dark-field (**b**) image of proton heated foam with 90 ps delay between the short-pulse laser hitting the aluminum foil for proton acceleration and the X-rays arriving at the sample. The proton beam heating the sample is incident on the left side of the images. The angled and striped line in (**a**) is a result of a non-sensitive gap in the detector interfering with image reconstruction. (**c**) shows a lineout of the dark-field data (raw data in pale red), smoothed by a running average with a window-size of 100 μm (red). For comparison, the cold foam’s dark-field signal is plotted in orange (extracted from the area marked in (**b**)). A detailed description of the transmission and dark-field calculation is given in the methods section.

### TMPTA foam

In the case of TMPTA foams, both the small, sub-micron spatial scale of the structures, as well as their stochastic distribution, preclude the use of conventional radiography to image the evolution of the structure towards a homogeneous distribution. Dark-field imaging is sensitive to small angle scattering on microstructures and as such is capable of detecting the presence of structured materials even if structure sizes are far below the imaging system’s resolution. As an example, Fig. 3 shows images (both transmission and dark-field) of a TMPTA foam target, obtained at a delay of $90\,\text {ps}$ after the pump pulse. For the transmission image, no effect of the proton heating is visible, since the absorption of the material does not change on scales that are resolvable. However, the dark-field image clearly shows a change in signal; the dark-field values approach the free-field value of 1 close to the proton source, indicating homogenized material, compared to approximately 0.8 in the structured (cold) foam far away from the proton source. The shape of the region with dark-field signals higher than the cold foam’s signal reflects the characteristic distribution of protons accelerated by the TNSA mechanism. Protons accelerated along the normal direction of the thin aluminum foil (left edge of Fig. 3) are more energetic and penetrate deeper into the sample, while those at increasing angles have lower energies and are stopped earlier. Due to the proton spectrum’s shape, most of its energy is deposited near the incident foam border and progressively less at greater depths. By imaging the entire foam sample, a range of different heating conditions are probed. A lineout through the centre of the heated area is plotted in Fig. 3c. The DF-signal decreases from a value around 1 (indicating an unstructured density distribution) at the beginning of the foam sample over about 500 micrometers to the value obtained from the cold (i.e. structured) sample.

**Fig. 4:**
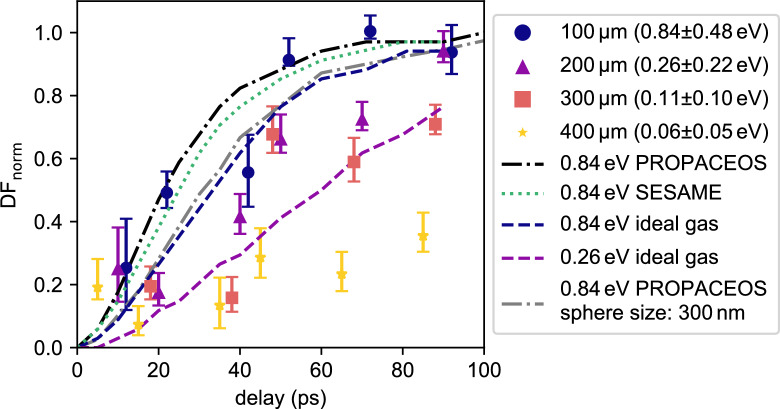
Displayed are the experimentally determined normalized dark-field values at different positions in the foam sample at different probe delays. These data points are compared with simulated loss of dark-field values, which are based on 1D hydrosimulations of spheres with $200\,\text {nm}$ radius. The simulations with PROPACEOS equation of states (EOS) and ideal gas EOS have been performed with HELIOS, the simulation with SESAME EOS was performed with FLASH.

By variation of the delay between pump and probe laser, the temporal evolution of the dark-field signal at selected positions can be observed. This is shown in Fig. 4 for positions along the center line, at distances from the front surface of the sample of 100, 200, 300 and 400 μm, respectively. The values plotted are the normalized dark-field values such that $\mathrm {DF_{norm}} = 0$ is the signal from the structured foam, and $\mathrm {DF_{norm}} = 1$ the signal of an unstructured, i.e. homogeneous density distribution. The delay plotted in Fig. 4 is the time since the foam has reached its maximum temperature. In practice, this corresponds to the pump-probe delay minus the time required for the protons to travel from the aluminum foil to the target and deposit their energy. Section Modeling elaborates on the estimation of this time.

At the position closest to the proton source (100 μm), $\mathrm {DF_{norm}}$ rises to approximately 50 % by 20 ps and reaches around 100 % after 60 ps. At 200 μm already, the rise is markedly slower, reaching 50 % after 40 ps and 100 % after 90 ps, respectively. This trend continues for the deeper positions of 300 μm and 400 μm, with $\mathrm {DF_{norm}}$ not even reaching values close to 1 within the maximum time delay of 100 ps.

From Fig. 3c, dark-field signal fluctuations on a larger spatial scale become apparent. While small scale noise can primarily be attributed to photon noise, broader modulations still persist after applying a moving average. These large-scale fluctuations suggest the presence of systematic artifacts, potentially stemming from grating defects or varying foam thicknesses. In order to account for both effects, the error bars in Fig. 4 incorporate the standard error and the variation introduced by different smoothing window sizes (50 μm–150 μm). Additionally, the error bars also include the contribution from shot-to-shot variations (detailed explanation in the methods section).

## Modeling

### Foam heating

**Fig. 5:**
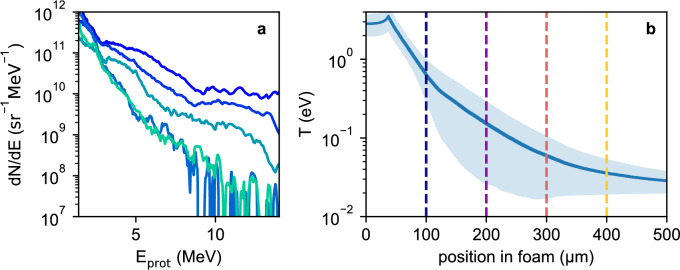
Spectrum for several diagnostic shots (in different colors) of the proton beam are shown in (**a**). The modeled temperature distribution within the foam target after being fully heated by the proton beam is displayed in (**b**). The confidence interval indicates the temperature uncertainty due to shot-to-shot fluctuations of the measured proton spectra (**a**). Vertical dotted lines indicate the positions, at which DF-values are measured for the plot in Fig. 4.

As the protons penetrate the foam samples, they are slowed down by Coulomb collisions, and thus deposit energy volumetrically in the sample material. Given the protons’ energy distribution (see Fig. 5a) and their angular divergence obtained from dedicated measurements with no sample, most of the energy is deposited at the sample front, decreasing with depth and distance to the source. Utilizing proton stopping powers from SRIM^21^, we have conducted raytracing simulations to calculate the time-dependent proton energy deposition within the foam target. The temperature is then calculated using the equation of state (EOS) from PROPACEOS^22^ and plotted in Fig. 5b. Since it is not possible to measure proton beam properties while heating a foam sample, repeatable conditions from shot to shot have to be assumed in order to assign an initial temperature to different locations within the sample. However, the proton spectra plotted in Fig. 5a show fluctuations in the energy distribution for different shots. The resulting shot-to-shot temperature uncertainties are indicated by the confidence interval in Fig. 5b.

The ray tracing simulations are additionally used to calculate the temporal evolution of the foam temperature, resulting from the dispersion of the proton pulse due to its broad energy distribution. After the pump pulse irradiates the aluminum foil, it takes roughly 18 ps at 100 μm and 25 ps at 400 μm to reach full heating. The effective heating phase is approximately 10 ps long, independent of the position within the foam target.

### Foam homogenization

To better understand the homogenization behavior of the foam, a simplified simulation model was developed. The internal structure of the chemically produced TMPTA foam was characterized using scanning electron microscopy (SEM), revealing an inhomogeneous network of small, clustered features surrounded by empty space (Fig. 1b). While a hydrodynamic simulation of a full 3D representation would require high-resolution nano-CT imaging and immense computational demands, a reduced model of randomly placed solid TMPTA spheres (enforcing a mean density of 250 mg/cm$^3$) was adopted. One-dimensional hydrodynamic simulations of these spheres were performed using HELIOS^22^ and FLASH^23^. To compare these results with the experimentally observed dark-field (DF) signal, wave-field propagation simulations developed at ECAP^24^ transform the resulting density distribution to a DF value. A more detailed description of this model can be found in the methods section.

Figure 4 compares these simulation results with the experimental data. The simulation was carried out with spheres of 200 nm radius (averaged over structure size distribution from SEM images). PROPACEOS (C_5_H_4_O_2_)^22^ and SESAME (7550 Mylar)^25^ have been used as equation of states. The simulations are performed at 0.84 eV, corresponding to the temperature 100 μm inside the target. Both FLASH (SESAME) and HELIOS (PROPACEOS) predict an increasing dark-field value that is in agreement with the experimental observations. Simulations with tabulated EOS at even lower temperatures were not possible, since both hydrocodes are not capable of dealing with material at solid-state conditions (which is modeled by the EOS table with negative ion pressure). To approximate the DF signal evolution at these lower temperatures, additional HELIOS simulations with an ideal gas EOS were performed. Although less accurate physically, they allow to explore qualitative trends. At 0.84 eV, the ideal gas simulation shows a slightly smaller slope in the signal. This same trend can be observed when comparing the 0.26 eV simulation with the experimental data at 200 μm within the target.

To test the validity of choosing a sphere radius of 200 nm, a simulation with a sphere size of 300 nm has been conducted. While the calculated DF signal differs and the results with the 200 nm sphere size fit the experimental values better, the impact of the sphere size on the simulation result is small.

## Summary and outlook

We presented homogenization evolution measurements of 3D printed and chemical foams at different initial temperatures. The evolution of the 3D printed structure was resolved directly using X-ray absorption imaging, demonstrating the capabilities of X-ray radiography to observe this new class of targets. The microstructural changes in the chemical foam samples were observed using grating-based X-ray dark-field imaging. Hydrodynamic simulations in combination with wave-field propagation of the simulated electron density distributions clearly link the measured signal to these microstructural changes of the heated foam. The good agreement of the FLASH and HELIOS simulations with the experimental data at 0.84 eV shows the effectiveness of the model. Notably, the experimental observations at lower temperatures provide valuable insight into foam behavior in regimes that remain challenging to model with hydrosimulations.

The main advantage of imaging with grating-based dark-field imaging is the decoupling of spatial resolution of the imaging system from the typical length scales of microstructures probed. By selecting appropriate interferometer parameters, changes in microstructure for a variety of samples can be imaged over a large field of view. Many of the remaining challenges stem from the X-ray source; shot-to-shot variations of source size, spectrum and brightness degrade reconstructed dark-field images. Furthermore, ejected debris from the backlighter and low laser repetition rates prevent taking cold images of each sample that would allow detecting and correcting differences in sample properties. Higher repetition, brighter X-ray sources such as X-ray free-electron lasers in combination with a grating setup specifically optimized for dark-field measurements promise further improvements in obtained data quality. X-ray dark-field imaging may thus establish itself as a powerful diagnostic for probing previously inaccessible features of microstructured targets, thereby advancing their fundamental understanding.

## Methods

### X-ray imaging and talbot interferometry

X-ray imaging is enabled by a laser-driven X-ray backlighter source, driven by the second high-energy short laser pulse, focused to a spot size of $\approx \textrm{5}$ μm (FWHM) onto a tungsten wire with 5 μm diameter. The energetic electrons generated in the relativistic laser-matter interaction produce both characteristic line emission and bremsstrahlung within the wire^26^. As the duration of this X-ray burst is shorter than the hydrodynamic expansion of the wire, the source size is defined by the wire dimension. X-ray images are acquired using the charge integrating, hybrid pixel JUNGFRAU^27^ detector, protected by an EMP protective housing^28^. The detector has a 320 μm thick silicon sensor with a pixel pitch of 75 μm. With this sensor thickness, X-ray absorption is close to 100 % for photon energies up to $10\,\text {keV}$^29^, making it ideally suited for the main imaging energy around 9.5 keV defined by the backlighter spectrum and grating interferometer. The sensor consists of a total of eight tiles arranged in four columns and two rows. A non-sensitive gap is present, where the tiles are butted together. The detector is placed at a distance of $585\,\text {mm}$ from the backlighter source, the samples at $31\,\textrm{mm}$, achieving a magnification of about $18.9$. A strong magnet and several filters — kapton, and a combined $2\,\textrm{mm}$ of beryllium — prevent debris and charged particles created in the laser-matter interaction from reaching the detector.

In order to obtain dark-field images, two gratings forming a Talbot-interferometer are placed in the imaging path. In this scheme, a phase grating G1, placed at $220\,\textrm{mm}$ from the source, imprints a periodic pattern onto the wave front. Since G1 is designed to be $\pi$-shifting at the main imaging energy of $9.5\,\text {keV}$, the Talbot pattern has a period equal to half the magnified grating period of $10.6$ μm. This pattern is repeated at the so-called Talbot distances downstream of G1 due to propagation effects^30,31^. Since the period of typical Talbot patterns is below the resolution limit of common X-ray detectors, the second grating G2, an absorption grating with a period of $9.5$ μm and a gold absorber height of about $70$ μm, is placed at $395\,\textrm{mm}$ from the source to sample the Talbot pattern. The superposition of the periodic grating with the Talbot pattern creates a Moiré pattern with a tunable period on the order of several pixels. This pattern is therefore directly resolved. Objects within the interferometer alter the wave front and thereby the Talbot pattern as well as the Moiré pattern by shifting the positions of fringes or reducing their contrast. This Moiré imaging scheme enables single-shot X-ray phase contrast imaging^32^ which is required due to the destruction of the samples with each shot.

The dark-field signal in particular arises from samples where typical structure sizes are below the resolution limit of the detection system and in general depends on parameters of the Talbot interferometer, the photon energy of the X-rays, and setup geometry via the correlation length^33–35^

1 $$\begin{aligned} \xi = \frac{\lambda d_{\overset{}{\text {G1G2}}}}{p_{\overset{}{\text {G2}}}} \left( 1-\frac{d_{\overset{}{\text {SG1}}}}{d_{\overset{}{\text {G1}}}} \right) . \end{aligned}$$

Here, $\lambda$ is the photon wavelength, $d_{\overset{}{\text {G1G2}}}$ the distance between G1 and G2, $p_{\overset{}{\text {G2}}}$ the period of G2, $d_{\overset{}{\text {SG1}}}$ the distance between sample and G1 and $d_{\overset{}{\text {G1}}}$ the distance between source and G1. If the projected electron density contains structures of sizes similar to the correlation length and with sufficient amplitude, a dark-field signal is expected^36^.

### Dark-field image reconstruction and background correction

**Fig. 6:**
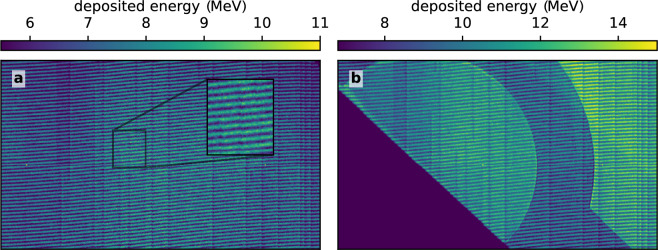
Raw detector images before the reconstruction. (**a**) shows a reference measurement including a zoomed-in view of the Moiré fringe pattern. (**b**) presents an object measurement.

The dark-field image is retrieved using the Moiré pattern from two distinct images; one reference image (denoted by a ”ref” index in the following), acquired with no object and one object image (denoted by a ”obj” index in the following). Example object and reference images are shown in Fig. 6.

The pixelwise reconstruction of transmission T, dark-field $\text {DF}$ and the differential phase is performed using Fourier analysis^32,37,38^. First, the ref and obj images are transformed to frequency space using 2D Fourier transforms. The Moiré pattern, being a periodic pattern, introduces a peak at its characteristic frequency, referred to here as the first Fourier order and denoted by $\mathfrak {F}_{1}$. A second peak is present around the zero frequency. This peak contains the attenuation information and is called zeroth Fourier order $\left( \mathfrak {F}_{0} \right)$ in the following. Applying appropriate filters^38^ to both orders and therefore separating first- and zeroth-order peaks allows deconvolution of the Moiré pattern from the standard intensity image information. The transmission and dark-field images are reconstructed from the separated peaks via

2 $$\begin{aligned} \text {T} = \frac{\left| \mathfrak {F}_{0,\, \text {obj}}^{-1} \right| }{\left| \mathfrak {F}_{0,\, \text {ref}}^{-1} \right| }, \hspace{0.25cm} \text {DF} = \frac{\left| \mathfrak {F}_{1,\, \text {obj}}^{-1} \right| }{\left| \mathfrak {F}_{0,\, \text {obj}}^{-1} \right| } \mathrel {\Bigg /} \frac{\left| \mathfrak {F}_{1,\, \text {ref}}^{-1} \right| }{\left| \mathfrak {F}_{0,\, \text {ref}}^{-1} \right| }= \frac{\left| \mathfrak {F}_{1,\, \text {obj}}^{-1} \right| }{\left| \mathfrak {F}_{1,\, \text {ref}}^{-1} \right| }\cdot \frac{1}{\text {T}}\,. \end{aligned}$$

In Fig. 2, the expression

3 $$\begin{aligned} 2 \cdot \frac{\left| \mathfrak {F}_{1,\,m}^{-1} \right| }{\left| \mathfrak {F}_{0,\,m}^{-1} \right| }=V_m,\quad m\in \{\text {ref}, \text {obj}\}. \end{aligned}$$

is called visibility^37^. The visibility is a measure of contrast of the Moiré pattern and dark-field signal can be interpreted as a loss of visibility due to the object. Similar to the transmission, dark-field values lie between 0 and 1, where 1 means no dark-field signal and 0 means maximum dark-field or complete loss of visibility.

**Fig. 7:**
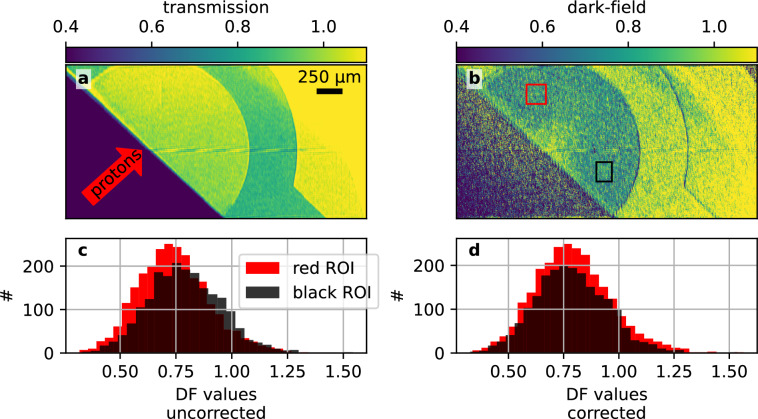
Transmission and dark-field images of a heated foam target. (**a**) Shows a transmission image with a gradient due to the pump laser used for TNSA. (**b**) shows the dark-field image with two ROIs where unheated foam is present. The striped horizontal line seen in (**a**) and (**b**) is caused by the non-sensitive gap in the detector. (**c**) shows the distribution of dark-field values before the correction in these ROIs. Shown in (**d**) is the distribution of dark-field values in the same ROIs after the dark-field image is background corrected.

In the presented experiment, the aluminum foil serving as the proton source also emits X-rays, introducing additional complexity into the reconstruction process. The X-rays from this secondary source do not follow the Talbot interferometer, since they are not emitted at the backlighter source position. Hence, they do not contribute to the formation of the Moiré pattern. Instead, they are seen as a varying intensity pattern in the object measurements. Such background patterns typically vary slowly and therefore have predominantly low-frequency components that are contained within the zeroth Fourier order of the object image $\left( \mathfrak {F}_{0,\, \text {obj}}^{-1}\right)$.

The background pattern therefore introduces a gradient into the dark-field images, since the zeroth-order peak is used in dark-field reconstruction (c.f. Fig. 3). This gradient affects absolute dark-field values and must be corrected if dark-field images from different shots are compared. Assuming that the object absorbs X-rays on average uniformly and that the source brightness differences between shots are mostly global changes in brightness, the background source causes an additive pattern $G$ within the reconstructed transmission image that can be removed via

4 $$\begin{aligned} T_\text {corrected} = T - G. \end{aligned}$$

An example of such a gradient is shown in Fig. 7a. For the presented data, a two-dimensional polynomial of second degree (6 parameters) consistently models the pattern caused by the background source within the foam in the transmission image, allowing the retrieval of the pattern $G$ via this fit. Replacing the standard transmission image in the dark-field reconstruction in Fig. 2 with the gradient corrected transmission Fig. 4 results in a gradient correction for the dark-field image:

5 $$\begin{aligned} \text {DF} = \frac{\left| \mathfrak {F}_{1,\, \text {obj}}^{-1} \right| }{\left| \mathfrak {F}_{1,\, \text {ref}}^{-1} \right| }\cdot \frac{1}{T_\text {corrected}} \end{aligned}$$

An example correction procedure is shown in Fig. 7. Presented in Fig. 7a is the raw transmission image with a strong gradient from the lower to the upper part of the image. Using the described method, the gradient introduced into the dark-field via $\mathfrak {F}_{0,\, \text {obj}}^{-1}$ is removed and the dark-field image Fig. 7b is obtained. The distribution of dark-field values in the lower and upper part of the foam are presented before and after the gradient correction in Fig. 7c and d. Before the gradient correction is applied, values closer to the upper edge show lower signals; The additional sources decrease visibility, therefore creating an artificial dark-field signal not caused by the object. The gradient compensation reduces the difference between the cold foam dark-field values in the parts, reducing this effect. However, the correction also renders dark-field values outside the fit region meaningless, as the fit only accurately models the background gradients for the fit region, where the foam is present. Outside of this region, the fit diverges from the measured data and introduces even stronger deviations from the real signal. This effect can be seen towards the lower-left and lower-right edges of Fig. 7b.

Each object measurement is reconstructed with three distinct reference images, the corrections are applied and the resulting images are averaged. With this procedure, potential influences on the measured dark-field signal introduced by shot-to-shot differences in the X-ray backlighter source are mitigated. The remaining uncertainty due to shot-to-shot differences is estimated using an unheated foam measurement. The mean dark-field value of a region of unheated foam is determined for reconstructions with the three available references. The standard error across these mean dark-field values is added to the other uncertainties in Fig. 4.

### Homogenization model of foam

**Fig. 8:**
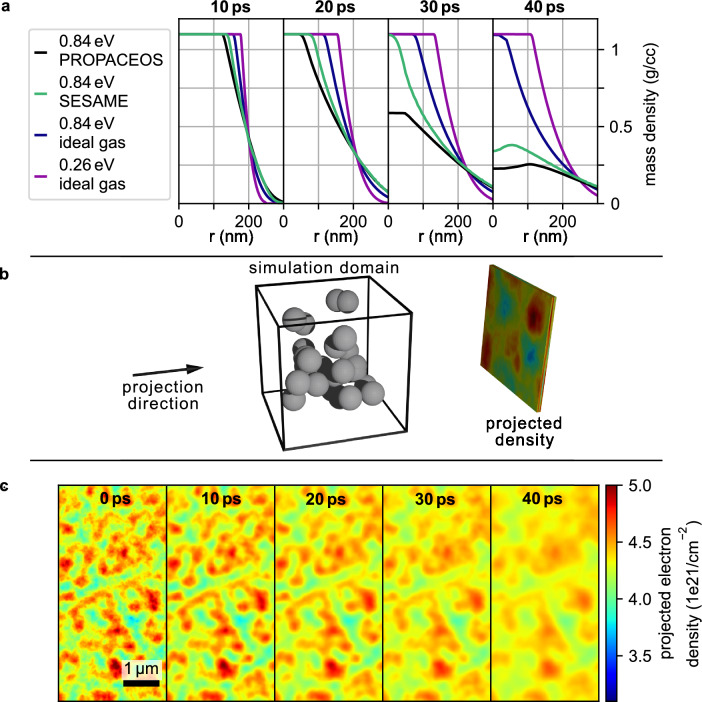
(**a**) displays the results of 1D hydrosimulations in spherical symmetry for different time steps. The simulations with PROPACEOS and ideal gas EOS are HELIOS simulations, while the simulation with SESAME EOS is performed with FLASH. (**b**) establishes the models setup. The spheres represent the density profiles of the 1D hydrosimulations, line-of-sight integration then reveals the projected density distributions that are shown at different time steps in (**c**). Here, the simulation at 0.84 eV with PROPACEOS EOS has been chosen as an example. This projected density evolution is used as the input for the wavefront simulations.

Based on the scanning electron microscope images of the foam, we have devised a simple model to mimic its structure. We use 200 nm radius spheres and randomly distributed them in a 3D simulation domain with a thickness of 500 μm to accurately account for the target thickness. The hydrodynamic evolution of an individual sphere is modeled in 1D (spherical geometry) using the simulation codes HELIOS^22^ and FLASH^23^ (see Fig. 8a). The corresponding radial density distributions obtained from the simulation then replace the solid spheres in the 3D domain. To reconstruct the projected electron density, the mass density is integrated along the simulation domain thickness, accounting for the distribution of sphere density profiles at each time step (see Fig. 8b). The temporal evolution is shown in Fig. 8c. As expected, a mass flux from areas with high density to areas with low density is present, as the foam homogenizes. Structures appear to grow in size and become less prominent, which in turn reduces the measured dark-field signal.

Accurately calculating a DF value from this density distribution is a challenging task. While several models exist to calculate the expected DF from the microstructure and statistical distribution within a sample^33,34,36^, they are generally hard to compute, and analytical expressions exist only for some select ensembles, such as collections of spheres in suspension^39^. Since the spheres in this model are expanding, it is not feasible to use these models to estimate the DF signals of foams during homogenization. Instead, wave field propagation simulations were conducted to gain insight into the dependence of the electron distribution on the resulting DF image.

We use a wave propagation code that was developed inhouse at ECAP^24^. It considers magnifications and gratings, and the resulting propagated wave is used to calculate a simulated object/reference image, assuming a simplified coherent source. These images are then combined in the reconstruction to obtain the expected changes in DF signal. To retrieve a DF signal, this code was adapted to accept 2D projected electron densities as input.

## Data Availability

The data that supports the findings of this study are available within the article and from the corresponding author upon reasonable request.
